# Open-Source Remote Gait Analysis: A Post-Surgery Patient Monitoring Application

**DOI:** 10.1038/s41598-019-54399-1

**Published:** 2019-11-29

**Authors:** Reed D. Gurchiek, Rebecca H. Choquette, Bruce D. Beynnon, James R. Slauterbeck, Timothy W. Tourville, Michael J. Toth, Ryan S. McGinnis

**Affiliations:** 1 M-Sense Research Group, University of Vermont, Burlington, VT 05405 USA; 2 Department of Orthopaedics and Rehabilitation, University of Vermont, Burlington, VT 05405 USA; 3 Department of Rehabilitation and Movement Science, University of Vermont, Burlington, VT 05405 USA; 4 Department of Medicine, University of Vermont, Burlington, VT 05405 USA

**Keywords:** Diagnostic markers, Rehabilitation

## Abstract

Critical to digital medicine is the promise of improved patient monitoring to allow assessment and personalized intervention to occur in real-time. Wearable sensor-enabled observation of physiological data in free-living conditions is integral to this vision. However, few open-source algorithms have been developed for analyzing and interpreting these data which slows development and the realization of digital medicine. There is clear need for open-source tools that analyze free-living wearable sensor data and particularly for gait analysis, which provides important biomarkers in multiple clinical populations. We present an open-source analytical platform for automated free-living gait analysis and use it to investigate a novel, multi-domain (accelerometer and electromyography) asymmetry measure for quantifying rehabilitation progress in patients recovering from surgical reconstruction of the anterior cruciate ligament (ACL). Asymmetry indices extracted from 41,893 strides were more strongly correlated (*r* = −0.87, *p* < 0.01) with recovery time than standard step counts (*r* = 0.25, *p* = 0.52) and significantly differed between patients 2- and 17-weeks post-op (*p* < 0.01, effect size: 2.20–2.96), and controls (*p* < 0.01, effect size: 1.74–4.20). Results point toward future use of this open-source platform for capturing rehabilitation progress and, more broadly, for free-living gait analysis.

## Introduction

The digital medicine revolution is driven by advances in wearable sensor technology and the algorithms for analyzing and interpreting their data. These mobile health technologies enable improved remote patient monitoring, personalized intervention, and could be used to provide improved continuity across care transitions. While transitional care has been recognized as a national priority^1^, and has been shown to improve outcomes, increase the efficient use of health care resources, and decrease health care costs^2,3^, current interventions are resource and personnel intensive^4^. Digital medicine innovations that harness existing cyber infrastructures, wearable sensors, and mobile devices may improve the efficiency and effectiveness of transitional care interventions. This approach could be transformative for a broad range of clinical domains, including for neurological^5^, musculoskeletal^6^, and mental health^7,8^ conditions.

Concerning current techniques for remote patient monitoring, physical activity is the most targeted health behavior, with a large commercial market. However, these measures are too general for most clinical applications as they provide minimal biomechanical or physiological insight at a joint- or limb-specific level. For both neurological and musculoskeletal disorders, physical activity (e.g. step counts) is often the primary outcome measure from free-living wearable sensor data^9,10^. In these clinical populations, traditional gait analysis provides far more valuable information concerning neuromusculoskeletal health and, in the aftermath of various clinical interventions, recovery of physical function^11^. However, these traditional assessments are constrained to specialized motion analysis laboratories which may not accurately reflect an individual’s free-living gait^12^. This motivates the pursuit of remote gait analysis techniques able to capture more clinically relevant biomechanics including quantitation of motor control indices (e.g. muscle activation patterns^13^) and musculoskeletal dynamics^14^.

To answer this unmet need, several groups have started to explore methods for tracking free-living gait biomechanics^12,15–21^. The general framework for remote gait analysis shared by these efforts has three steps: (1) identification of walking bouts, (2) stride detection, and (3) analysis. This approach has been used to detect and characterize bradykinesia in Parkinson’s disease patients^15^ and demonstrate that in-lab observations of gait speed^12^ and gait asymmetry^20,21^ differ from daily-life. The components of this framework reflect recent advances in wearable sensor-based activity identification^22^, event detection^23^, and biomechanical analysis^24–29^. The development and application of these solutions are multi-disciplinary efforts requiring research teams with expertise in medicine, data science, and engineering. Open-source solutions may allow analysis of wearable sensor data collected in free-living conditions by teams without expertise in signal processing or machine learning. This reduces barriers to applying digital health solutions for remote patient monitoring.

**Figure 1:**
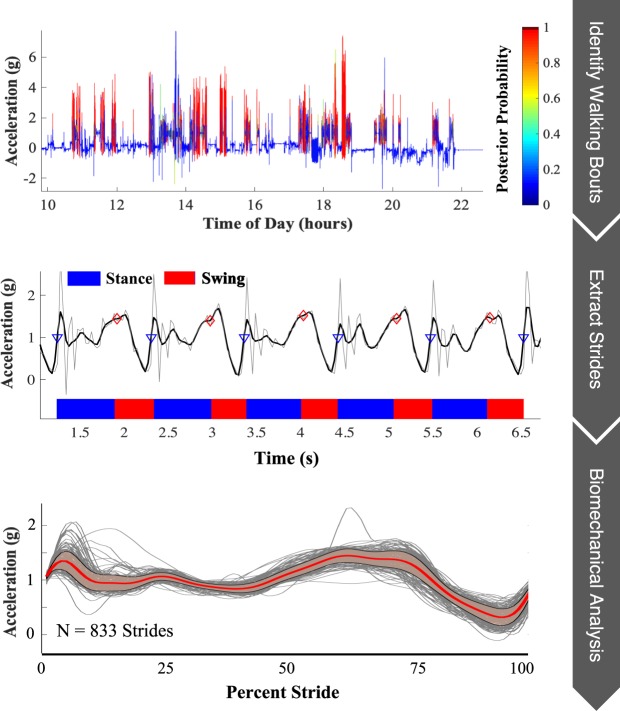
Graphical summary of the proposed remote gait analysis. The proposed approach is comprised of three basic steps: (1) walking bout identification, (2) stride extraction and gait phase segmentation, and (3) biomechanical analysis of individual strides.

To this end, we present an open-source analytical platform which captures this general framework (Fig. 1) and could be applied for remote biomechanical analysis of any task (see https://github.com/M-SenseResearchGroup/RemoteBMX). It has been designed with a modular structure to enable flexibility and to encourage improvements and customization from members of the scientific community. For example, customization could allow for the analysis of other tasks (e.g. stair ascent, running), the extraction of alternative clinical information, or the utilization of different wearable sensors. These modifications are dependent on the patient population being monitored.

To demonstrate how the platform may be tailored for a specific patient population, it was deployed as a rehabilitation monitoring application in patients recovering from reconstructive surgery of the anterior cruciate ligament (ACL-R). ACL-R is the most effective treatment for ACL rupture^30^. However, approximately 50% of patients who undergo ACL-R will go on to develop post-traumatic osteoarthritis (PTOA)^31^. Previous research suggests that altered gait biomechanics following ACL-R are responsible for this phenomenon^31,32^. Pathological gait is often characterized by inter-limb asymmetries which manifest early in the post-surgical period^33–35^, and develop into compensatory gait patterns over time^11,36^. It is imperative that these maladaptations are identified early so that corrective rehabilitative interventions can be pursued^37^. Thus, this application presents an ideal candidate for which to demonstrate the clinical utility of the proposed platform.

Platform modifications were context-specific and driven by the current understanding of the clinical problem. In the context of ACL-R, inter-limb gait asymmetry has been identified as a biomarker for recovery^33,34,38^. These asymmetries represent altered gait kinematics that are linked to PTOA-related knee joint contact forces in this population^11,39,40^. Quadriceps muscle dysfunction is common following ACL-R^13,38,41,42^ and may be responsible for the development of pathological gait. Thus, direct observation of asymmetric gait kinematics and quadriceps muscle activity in the early post-operative period could identify pathological gait and signal the need for remedial actions to prevent developing PTOA. However, algorithms for extracting this information from wearable sensor data captured in free-living conditions do not yet exist.

To this end, patients recovering from ACL-R present an ideal population for which to specify and test the use of the proposed platform. Our analysis was tailored for monitoring gait asymmetries relating to spatiotemporal variables, and kinematic and muscle activation time-series. Our novel analysis quantifies asymmetry in the following gait measures: duty factor (DF); mean surface electromyography (sEMG) amplitude of the rectus femoris during the stance and swing phase of gait; muscle activation time-series; as well as cranial-caudal (CC), medio-lateral (ML), and antero-posterior (AP) thigh acceleration time-series. A composite asymmetry score was also defined as the average of these seven asymmetries. We show the ability of these asymmetry indices to discriminate between patients at different time points in the recovery process and compared to healthy controls. Step count estimates from a commercially available activity monitor worn by some of the patients also enables a comparison of the proposed analysis to current techniques for monitoring recovery.

## Results

**Figure 2:**
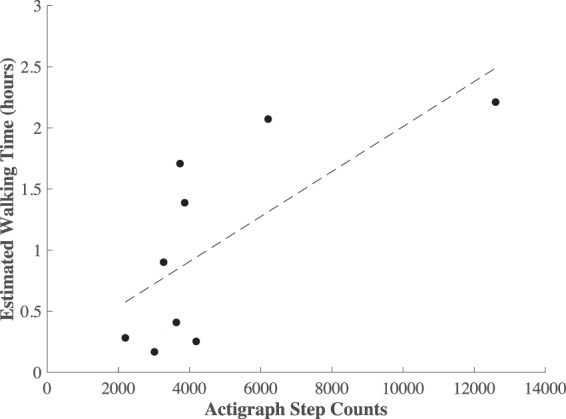
Scatter plot of the total time spent walking from the proposed method vs step counts estimated by Actigraph activity monitors.

**Figure 3:**
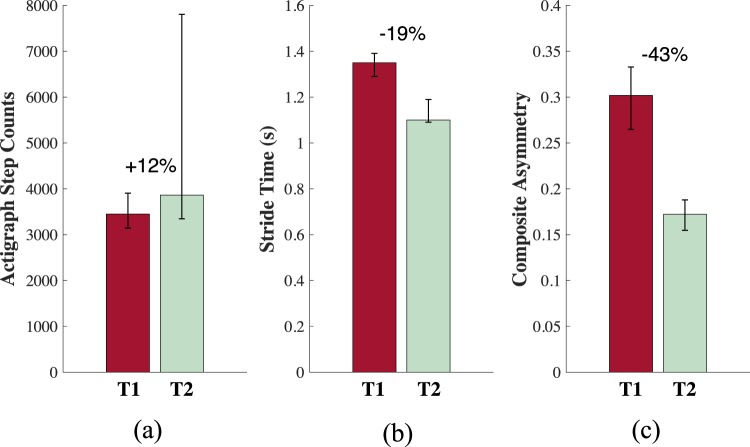
Percent difference in the median Actigraph step counts (**a**), strides times (**b**), and composite asymmetry scores (**c**) between the T1 (red) and T2 (green) groups. Error bars denote the 25^th^ and 75^th^ quantiles.

Subjects were categorized as either T1 (less than 6 weeks post-surgery), T2 (greater than 6 weeks post-surgery) or C (healthy controls). Data were collected for 20.24 ± 6.28 hours on average for each subject (except one T1 patient for whom no walking bouts met the criteria for analysis) and a total of 41,893 strides were analyzed (T1: 1,743 strides, T2: 9,616 strides, C: 20,939 strides). Estimated total time spent walking (hours) was strongly correlated (*r* = 0.71, *p* = 0.03) with Actigraph step counts (Fig. 2) but showed a stronger correlation with recovery time (*r* = 0.63, *p* = 0.08) than Actigraph step counts (*r* = 0.25, *p* = 0.52), although neither was statistically significant. Importantly, the composite asymmetry score (*r* = −0.87, *p* < 0.01) and stride time (*r* = −0.91, *p* < 0.01) were both strongly associated with recovery time (see Fig. 3).

Stride times and most gait asymmetry measures decreased across groups (i.e. T1 > T2 > C). Pairwise significant differences were found between C and T1 as well as T1 and T2 for stride times and all asymmetries (all with large effect sizes) except mean normalized sEMG which was trending towards significance (stance: *p* = 0.05, swing *p* = 0.12) (Table 1). All time-series (sEMG, CC, AP, ML), duty factor, and composite asymmetries were significantly different (*p* = 0.01) between C and T1, but only the CC acceleration time-series, duty factor, and composite asymmetries were significantly different between T1 and T2 (Table 1). There were no differences between C and T2 for any asymmetries.

**Table 1:** Comparison of daily average stride times and asymmetries.

	Mean (SD)			Pairwise Comparisons		
	T1	T2	C	C-T1	C-T2	T1-T2
ST	1.34 (0.08)	1.14 (0.07)	1.10 (0.05)	4.08**	0.34	2.70**
DF	0.13 (0.05)	0.04 (0.02)	0.03 (0.01)	4.20**	0.61	2.48**
EMG St	0.22 (0.12)	0.13 (0.03)	0.15 (0.04)	ANOVA *p* = 0.05		
EMG Sw	0.41 (0.14)	0.27 (0.04)	0.29 (0.12)	ANOVA *p* = 0.12		
EMG(t)	0.41 (0.10)	0.29 (0.11)	0.25 (0.07)	2.10**	0.52	0.07
AP(t)	0.32 (0.13)	0.10 (0.05)	0.07 (0.04)	3.57**	0.49	0.08
ML(t)	0.38 (0.13)	0.28 (0.04)	0.24 (0.06)	1.74**	0.51	0.08
CC(t)	0.20 (0.08)	0.06 (0.04)	0.03 (0.02)	4.19**	0.26	2.20**
Comp.	0.29 (0.05)	0.17 (0.03)	0.15 (0.03)	3.78**	0.65	2.96**

ST: Stride Time; units seconds. Duty Factor (DF), EMG Stance (EMG St), and EMG Swing (EMG Sw) asymmetry scores are the percent difference between the healthy and injured leg (i.e. 0.5 indicates that the between leg difference is 50% that of the healthy leg). EMG(t), AP(t), ML(t), and CC(t) are pattern asymmetries for the sEMG time-series and the antero-posterior, medio-lateral, and cranial-caudal thigh acceleration time-series respectively. Composite asymmetry score (Comp.) is the average value of the other seven asymmetry scores. Bold numbers in the pairwise comparisons are effect sizes (*p ≤ 0.05, **p ≤ 0.01) and non-bold numbers are the p values for non-significant pairwise differences.

**Figure 4:**
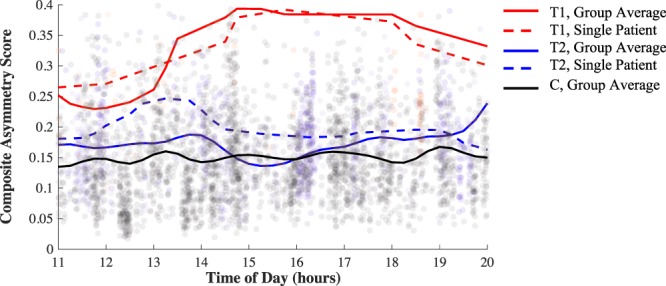
Composite asymmetry score throughout the day (averaged over every 15-minute bin) for a patient with longitudinal observations: 2.1 weeks post-surgery (red dashed line) and 19.1 weeks post-surgery (blue dashed line). The solid lines illustrate the average trends for the T1 (red), T2 (blue), and C (black) groups. The longitudinal patient’s data was not included in the group means.

The proposed framework enables the investigation of gait biomechanics continuously throughout the day. To demonstrate the utility of this application, composite asymmetry scores averaged over each 15-minute bin are illustrated in Fig. 4 for one patient with longitudinal observations at about 2 weeks post-surgery (red dashed line) and again 17 weeks later (blue dashed line). The solid lines illustrate the average trend of the other groups for comparison (T1: red, T2: blue, C: black).

## Discussion

Herein, we present a new computational platform that enables multi-modal gait analysis in free-living conditions based on data from commercially available wearable devices. We demonstrate the utility of this platform in a sample of patients recovering from ACL-R. Results suggest that platform-derived gait measures agree with gold standard actigraphy but better discriminate between patients’ gait at different time points in the recovery process. Similarly, platform-derived measures are able to detect gait differences between patients at different stages of recovery, and with large effect sizes. We further discuss the implications of these results and how this new platform could be deployed for remote gait analysis in a variety of clinical populations.

Currently, actigraphy technologies are broadly used for characterizing free-living physical activity and gait^9,10^. The statistically significant association we observe (Fig. 2; *r* = 0.71, *p* = 0.03) between actigraphy-derived daily step counts and platform-derived walking time suggest that the proposed analysis platform is valid for capturing free-living physical activity. Similar findings of agreement between different indices of physical activity have been reported elsewhere^9^. Although the correlation between recovery time and the platform-derived measure of walking time (*r* = 0.63, *p* = 0.08) appears stronger than that for actigraphy-derived step counts (*r* = 0.25, *p* = 0.52), neither was statistically significant, which also agrees with previous work^9^. This finding is intuitive as there are a variety of extraneous variables that affect daily physical activity and have nothing to do with rehabilitation progress (e.g. weather, day of the week). These results suggest a fundamental limitation of physical activity indices for monitoring rehabilitation progression. The vast majority of research investigating pathological biomechanics characteristic of gait following ACL-R suggests the existence of more sensitive metrics including quadriceps activation^43^, ground reaction force^33^, and joint work^34^. However, these metrics currently require laboratory-based methodologies that are not widely transferable to clinical use.

The proposed platform answers this unmet need by capturing more subtle biomechanical changes in gait associated with recovery under free-living conditions. Our results demonstrate that platform-derived stride times are more strongly associated with recovery time (*r* = −0.91, *p* < 0.01) than both actigraphy-derived step counts (*r* = 0.25, *p* = 0.52) and platform-derived estimates of total walking time (*r* = 0.63, *p* = 0.08). These results are supported by previous work where decreased walking speeds have been associated with decreased joint health following ACL-R^44^ (stride times are associated with walking speed^45^). These results provide additional evidence in support of the utility of platform-derived measures of free-living gait biomechanics.

Stride time, walking speed, and other spatiotemporal parameters are informative biomechanical measures related to recovery in this population, and more broadly are important indicators of mobility impairment^12,27,28,44^. Nevertheless, a need has been recognized for more sensitive biomarkers. For example, it has been shown that spatiotemporal *symmetry* may manifest even in the presence of a true gait abnormality^46^. Further, research suggests the pursuit of metrics that characterize the *full waveform pattern* of mechanical variables throughout the gait cycle^47^. In light of these results, the proposed platform has been designed to capture additional measures that may be more indicative of, and sensitive to, rehabilitation progress. Figure 3 and Table 1 report results from our efforts to define a novel gait asymmetry analysis that incorporates temporal (duty factor), kinematical (ML, AP, CC acceleration time-series), and neuromuscular (sEMG Swing, sEMG Stance, and sEMG time-series) measures. The effect sizes of the differences between groups were largest for the asymmetries relating to kinematic measures and duty factor. The mean normalized sEMG during stance and swing asymmetries were the only variables for which no significant difference was found, which may reflect increased within subject and group variance. The composite measure that captures asymmetries within each of these domains demonstrates significant differences between T1 and T2/C with large effect sizes (*d* = 2.96 and *d* = 3.78 respectively) suggesting that this measure may be useful for tracking biomechanical changes associated with rehabilitation progress.

To this end, we further examine the association of these measures of free-living gait biomechanics with time since surgery. Both stride time and our new composite asymmetry score present strong relationships with recovery time (*r* = −0.91, *p* < 0.01 and *r* = −0.87, *p* < 0.01 respectively), and are noticeably larger than that observed for any gross index of physical activity. Further, when comparing T1 and T2, the largest effect sizes were observed for the composite asymmetry (*d* = 2.96) which was also responsible for the largest percent difference between T1 and T2 (−43%) when compared to both stride time (−19%) and step counts (+12%) (Fig. 3). This suggests that these more detailed biomechanical measures may provide increased sensitivity to recovery time in this population and thus may be suitable candidates to pursue in developing novel *digital biomarkers* for tracking rehabilitation progress and gait asymmetries that have pathological consequences^33,34^. The proposed asymmetry analysis also provides the clinician with insight into patient-specific adaptations and their particular mechanistic origin since it captures indices of both muscle activation and limb kinematics.

Having established the improved association between free-living measures of gait asymmetry and recovery time, we further examine how this new measure changes over the entire wear time for patients monitored in this study. Figure 4 reports the composite asymmetry score captured during every gait bout identified between 11 am and 8 pm for a single patient with longitudinal observations and with the group average trend for subjects in the T1, T2, and C groups for comparison. The difference between groups based on asymmetry magnitudes alone reflects the same general pattern observed in Table 1, namely a convergent trend toward decreased gait asymmetries with increased recovery time. However, Fig. 4 provides additional insight whereby gait asymmetries appear more variable throughout the day for the early post-surgery time point when compared to the later time point and that of the control group. This supports the need for remote gait monitoring, as this variability would not be captured in a single gait assessment. Further investigation is necessary to understand the origins of this observation which may, for example, indicate an increased susceptibility to fatigue early in rehabilitation. The similar trends observed between the dashed lines (single patient, different time points) and the solid lines (average of all other patients within respective group) of like colors suggests the results of our cross-sectional design may mirror what would be seen in a longitudinal study. Further investigation using a longitudinal design is necessary to confirm this conjecture.

Far fewer strides were identified and analyzed for patients in the T1 group (≈350 strides/day per patient) than for those in the T2 (≈1,600 strides/day per patient) and C (≈1,310 strides/day per patient) groups. One explanation is that the T1 patients simply walk less, which is also supported by the Actigraph step counts. Intuitively, this may reflect a natural tendency for an individual to avoid an activity like walking which loads the recently reconstructed knee. Despite the difference in the number of strides analyzed for the T1 group compared to T2/C, we see this as an acceptable limitation as the availability of gait biomechanics characterizing even 350 strides per day is already a substantial improvement over the current standard which often yields fewer observed strides and with limited ecological validity.

**Figure 5:**
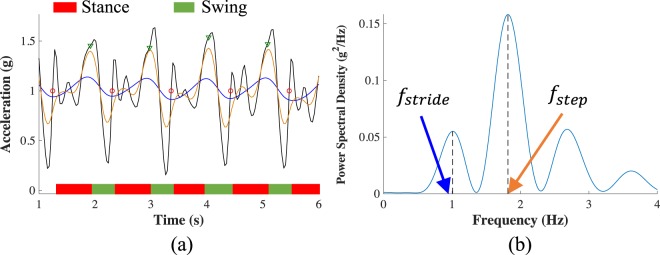
Stride detection and segmentation example. (**a**) Foot contact (red circles) and foot off (green triangles) events are identified using the CC-axis accelerometer time-series lowpass filtered with a 5 Hz cutoff (black trace) and with cutoff frequencies equal to the approximate step frequency (orange trace) and stride frequency (blue trace). Step and stride frequencies are approximated using the power spectral density of the raw accelerometer signal (**b**).

In the approach to remote patient monitoring proposed herein, we chose sensor locations that would minimize user burden while also providing the kinematic and muscle activity data key to the presented analysis. A minimum of one sensor per leg was required to extract inter-leg kinematic and muscle activation asymmetries. Future work could consider additional sensor locations in an effort to provide improved clinical utility. For example, if the muscle activity from a quadriceps antagonist (e.g. a knee flexor) were available it may provide insight into co-contraction indices and would perhaps make the knee extensor muscle activation time-series more interpretable. Inclusion of a sensor on the shank could allow for the extraction of knee joint kinematics^48,49^ that could add additional information indicative of recovery. However, current hardware constraints on the capacity of on-board memory and battery prevented use of gyroscope data and required a relatively low accelerometer sampling frequency (31.25 Hz) in order to enable sEMG data collection over the recording durations considered herein. While higher sampling frequencies and additional sensor modalities would be useful, accelerometer data recorded at 31.25 Hz is likely sufficient for capturing the segment kinematics of interest which is supported given the accelerometer signal power spectrum (see Fig. 5) and the fact that traditional laboratory-based gait analysis often employs cutoff frequencies ≤ 8  Hz for lowpass filtering kinematic data^45,50,51^.

The results presented herein demonstrate the clinical utility of the proposed analysis platform for remote monitoring of patients recovering from ACL reconstruction surgery. Our analysis describes an unprecedented evaluation of muscle activation concurrently with kinematical time-series during free-living gait. These constitute a novel asymmetry analysis which presents a substantial improvement over current techniques utilized for remote monitoring. Further investigation is necessary to fully realize the practical application of the proposed approach for continuous patient monitoring with a larger sample, longitudinal design, and more frequent observation (i.e. more than just two time points) during the recovery process. The underlying MATLAB code has been open-sourced and structured in a modular fashion so that users can easily add/edit functionality for their specific use case. For example, different activity classification models or stride segmentation algorithms that incorporate data from other wearable sensors located on different body segments could be inserted that are more precisely tuned for a given patient population. There are certainly algorithmic improvements and additions that could be made for the proposed remote gait analysis such as automatic anatomical calibration or estimation of other biomechanical variables. The modularity of the platform is intended to promote these improvements so that the platform may serve as a basis upon which to build a comprehensive approach for remote gait analysis. This contribution comes at a critical time in the use of wearable sensors for providing free-living patient monitoring capabilities. An open-source platform will promote cross-disciplinary efforts to further advance remote patient monitoring paradigms and digital medicine. Future research should investigate similar applications in other clinical contexts and begin the difficult task of understanding how to translate the detailed, big-data evaluations enabled by continuous monitoring paradigms into optimizing patient-specific interventions.

## Methods

### Study design

Our system was deployed to evaluate gait in three groups of subjects: (1) subjects less than six weeks after ACL-R (T1: 3 male, 3 female, recovery time (mean ±SD) = 2.1 ± 1.6 weeks, age = 26 ± 11 yo, height = 1.74 ± 0.11 m, mass = 70.52 ± 16.21 kg), (2) subjects at least six weeks after ACL-R (T2: 2 male, 4 female, recovery time = 17.2 ± 2.0 weeks, age = 26 ± 6 yo, height = 1.70 ± 0.13 m, mass = 77.82 ± 15.44 kg), and (3) healthy controls (C: 8 male, 8 female, age = 23 ± 5 yo, height = 1.74 ± 0.11 m, mass = 70.51 ± 13.17 kg). Each subject wore a single sensor (BioStamp, MC10 Inc., Lexington, MA) on the muscle belly of the rectus femoris of each thigh, which recorded tri-axial accelerometer (sampling frequency: 31.25 Hz, range: ± 16 g) and surface electromyography (sEMG) (sampling frequency: 250 Hz) data during daily life for one day. Daily step counts from a waist worn activity monitor (Actigraph, Pensacola, FL) were also available for nine ACL-R patients (T1: N = 4, T2: N = 5). These enable a comparison between the proposed analysis and current standards (e.g step counts^9^) in their respective sensitivity to patient recovery. This study was approved by the University of Vermont Institutional Review Board. All study activities were in accordance with the relevant guidelines and informed consent was obtained from all participants.

### Remote gait analysis platform

The general approach to biomechanical analysis of any task in daily life using the proposed platform takes on the following form: (1) activity identification, (2) event detection, and (3) analysis. The specifications for the current ACL-R patient monitoring application are described graphically in Fig. 1. This framework easily allows future users to make application-specific modifications to analyze different tasks (e.g. crutching, stair ascent) or analyze differently the same tasks presented in the current study. For monitoring ACL-R patients, the proposed analysis requires, at a minimum, raw accelerometer data to operate. Herein, we also consider sEMG data to provide a more complete picture of free-living gait biomechanics in this population.

### Identification of walking bouts

A support vector machine (SVM) binary classifier (Gaussian kernel) was used to identify walking bouts. The model was trained using annotated tri-axial accelerometer data from the healthy control group recorded during various activities including multi-speed walking and running over ground and on a treadmill, stair ascent and descent, crutching, sitting, standing, and lying down. Annotated walking, crutching, lying down, sitting, and standing data were also available from three ACL-R patients. Data from each activity were partitioned into four-second, non-overlapping windows. This yielded a total of 3,102 observations (1,318 walk; 1,784 not walk) for training the walking classifier.

The constant thigh relative sensor attitude was determined for each leg by considering accelerometer data during a standing calibration trial (as in^24^) and used to project raw accelerometer data onto the thigh-fixed cranial-caudal axis (referred to as *CC*_*i*_, *i* ∈{*R*, *L*}, *R*: right, *L*: left, directed proximally). The first principal component of acceleration in the transverse plane during each four-second window was assumed to coincide with the antero-posterior axis of the thigh (referred to as *AP*_*i*_, points anteriorly, see^48^), from which the medio-lateral axis (referred to as *ML*_*i*_, points laterally) can also be determined. Accelerometer data were projected onto these three anatomical axes and used to extract a total of 152 time and frequency domain features. Performance of the classifier was evaluated using leave-one-subject-out (LOSO) cross-validation where, for each iteration, data from one subject was removed for testing and a classifier was trained on the remaining data using only those features from the training set with a Davies-Bouldin index^52^ (*DBI*) less than two. From the LOSO validation, the classifier achieved 98.32% accuracy, 97.04% sensitivity, 99.27% specificity, and the area under the ROC curve (AUC) was 1.00. The classifier made no misclassification errors (100% accuracy) on the 132 observations from the three ACL-R patients during the LOSO validation, which supports its use to identify gait in this population.

Following this performance characterization, annotated data from all sixteen healthy controls and the three ACL-R subjects were used to train the classifier deployed for the proposed analysis. Davies-Bouldin feature selection (*DBI* < 2) indicated six features to use as input to the classifier: (i) average 25^th^ quantile of *CC*_*R*_ and *CC*_*L*_, (ii) average correlation between *CC*_*R*_ with *AP*_*L*_ and *CC*_*L*_ with *AP*_*R*_, (iii) correlation between *CC*_*R*_ with *CC*_*L*_, (iv) average skewness of *AP*_*R*_ and *AP*_*L*_, (v) average percentage of signal power contained below 0.25 Hz for *AP*_*R*_ and *AP*_*L*_, and (vi) average median value of *AP*_*R*_ and *AP*_*L*_.

These six features were extracted from each four-second window of raw accelerometer data collected during daily life from all subjects and were used as input to the SVM classifier to identify each window as *walking* or *not walking*. This enabled an estimate of the total amount of time spent walking which we used as an indicator of gross physical activity. Windows labeled as walking were aggregated into walking bouts if (1) at least two consecutive windows (8 seconds) were classified as walking, and (2) the posterior probability of the window’s walking classification was at least 0.8. This threshold was determined as the point that minimized the distance between the classifier’s ROC curve and the point (0,1) on the true positive rate-false positive rate plane. A very small subset (451 observations or approximately 0.19%) of bouts containing clipped sEMG (e.g. due to sensor delamination from skin) and/or accelerometer signals were removed.

All aspects of the walking classifier presented herein (feature extraction, training, validation, etc.) were performed using the MATLAB R2018a Statistics and Machine Learning Toolbox (Version 11.3) and our open-source platform specifically designed for wearable sensor-based activity identification. This platform streamlines the development of activity classifiers by enabling the building of population-specific feature sets, the extraction of novel features, feature manipulation (e.g. PCA, DBI), training and testing of various classification models, and automated leave-one-subject-out cross-validation with detailed error analysis. For more details see https://github.com/M-SenseResearchGroup/ActivityIdentification.

### Stride extraction and gait phase segmentation

Gait events were identified for each walking bout by considering the CC accelerometer signal passed through a bank of lowpass filters with cutoff frequencies at 5 Hz and the stride and step frequencies (*f*_*stride*_, *f*_*step*_). Stride and step frequencies were estimated for each walking bout from Welch’s power spectral density (PSD) of the signal (see Fig. 5). As illustrated in Fig. 5, local minima and maxima in the *f*_*stride*_- and *f*_*step*_-filtered signals indicate the initiation of the swing phase of gait (i.e. foot off). The first transition in the 5 Hz-filtered signal from below to above 1 g following foot off indicates the initiation of the stance phase of gait (i.e. foot contact). Constraints were placed on the stride time (0.91 s–1.57 s) and duty factor (DF, percentage of stride cycle spent in stance) (0.44–0.73) of identified strides to avoid accidental analysis of non-walking data^45^. To be considered for further analysis, an eight-second walking bout had to include at least two strides.

### Biomechanical analysis

Accelerometer and sEMG signals from each extracted stride were used to compute discrete biomechanical variables to evaluate gait. We quantify asymmetries in gait kinematics and muscle activity between legs during each stride using accelerometer data in each anatomical direction lowpass filtered at a frequency of 6 Hz (as per^51^) and the envelope of the sEMG data (computed as per^51^). These four time-series (sEMG, CC, AP, ML) were normalized by stride time so that each sample corresponds to a percentage of the gait cycle.

Eight indices of asymmetry (referred to as asymmetries) were computed for each eight-second walking bout. Three of these asymmetries (*a*_*k*_, *k* ∈{1, 2, 3}) were the relative difference between discrete biomechanical variables of each leg as per

1 $$\begin{array}{c}{a}_{k}=|\frac{{I}_{k}-{H}_{k}}{{H}_{k}}|\end{array}$$

where *I*_*k*_ and *H*_*k*_ are the values of discrete variable *k* for the injured and healthy leg respectively. The three discrete variables for this analysis were mean sEMG during stance, mean sEMG during swing, and DF. The other four asymmetries (*a*_*m*_) are defined as per

2 $$\begin{array}{c}{a}_{m}=\frac{1}{2}(1-{r}_{m}).\end{array}$$

where *m* ∈{sEMG, CC, AP, ML}, and *r*_*m*_ is the correlation coefficient of the ensemble means of like time-series between the injured and healthy legs. The final index of asymmetry was a composite asymmetry score equal to the mean of the aforementioned asymmetries.

### Statistical analysis

Outlier asymmetries were identified for each patient and removed after the biomechanical analysis as a final check to remove potentially errant data. A one-way analysis of variance (ANOVA) was used to compare the daily average of gait asymmetries and stride times between the three groups. Normality was checked using the Kolmogorov-Smirnov test. If the assumption of normality was violated, group distributions were compared using the Kruskal-Wallis test. If a significant difference was detected, post-hoc pairwise comparisons were made using Tukey’s honest significant difference criterion. Effect sizes (Cohen’s *d*) were computed where the ANOVA revealed significant differences and were interpreted qualitatively as weak (*d* < 0.25), small (0.25 ≤ *d* < 0.5), medium (0.5 ≤ *d* < 1.0), and large (*d* ≥ 1.0)^53^. The agreement between our estimate of gross physical activity (total time spent walking) and the Actigraph step counts estimate was evaluated using Pearson’s correlation.

To compare the sensitivity of physical activity with that of our composite asymmetry score and estimated stride times to time spent in recovery, we also determine the correlation between recovery time with composite asymmetry, stride time, Actigraph step counts, and estimated time spent walking for each ACL-R patient using Spearman’s rank correlation. The level of significance was set to 0.05 for all statistical tests.
